# Why do some inter-organisational collaborations in healthcare work when others do not? A realist review

**DOI:** 10.1186/s13643-021-01630-8

**Published:** 2021-03-22

**Authors:** Justin Avery Aunger, Ross Millar, Joanne Greenhalgh, Russell Mannion, Anne-Marie Rafferty, Hugh McLeod

**Affiliations:** 1 Health Services Management Centre, Park House, University of Birmingham, Birmingham, B15 2RT UK; 2 Sociology and Social Policy Department, University of Leeds, Leeds, LS2 9JT UK; 3 Florence Nightingale Faculty of Nursing, Midwifery and Palliative Care, King’s College London, London, SE1 8WA UK; 4 Population Health Sciences, University of Bristol & NIHR Applied Research Collaboration West, 9th Floor, Whitefriars, Lewins Mead, Bristol, BS1 2NT UK

**Keywords:** Collaboration, Partnership working, Integration, Healthcare, Improvement, Realist review, Realist synthesis, Context, Programme theory, Implementation

## Abstract

**Background:**

Inter-organisational collaboration is increasingly prominent within contemporary healthcare systems. A range of collaboration types such as alliances, networks, and mergers have been proposed as a means to turnaround organisations, by reducing duplication of effort, enabling resource sharing, and promoting innovations. However, in practice, due to the complexity of the process, such efforts are often rife with difficulty. Notable contributions have sought to make sense of this area; however, further understanding is needed in order to gain a better understanding of why some inter-organisational collaborations work when others do not, to be able to more effectively implement collaborations in the future.

**Methods:**

Realist review methodology was used with the intention of formulating context-mechanism-outcome configurations (CMOCs) to explain how inter-organisational collaborations work and why, combining systematic and purposive literature search techniques. The systematic review encompassed searches for reviews, commentaries, opinion pieces, and case studies on HMIC, MEDLINE, PsycINFO, and Social Policy and Practice databases, and further searches were conducted using Google Scholar. Data were extracted from included studies according to relevance to the realist review.

**Results:**

Fifty-three papers were included, informing the development of programme theories of how, why, and when inter-organisational collaborations in healthcare work. Formulation of our programme theories incorporated the concepts of partnership synergy and collaborative inertia and found that it was essential to consider mechanisms underlying partnership functioning, such as building trust and faith in the collaboration to maximise synergy and thus collaborative performance. More integrative or mandated collaboration may lean more heavily on contract to drive collaborative behaviour.

**Conclusion:**

As the first realist review of inter-organisational collaborations in healthcare as an intervention for improvement, this review provides actionable evidence for policymakers and implementers, enhancing understanding of mechanisms underlying the functioning and performing of inter-organisational collaborations, as well as how to configure the context to aid success. Next steps in this research will test the results against further case studies and primary data to produce a further refined theory.

**Systematic review registration:**

PROSPERO CRD42019149009

**Supplementary Information:**

The online version contains supplementary material available at 10.1186/s13643-021-01630-8.

## Background

Inter-organisational collaboration continues to be promoted as a cure-all for the many ills that ail modern healthcare systems and the broader public sector [1]. Defined as ‘a mutually beneficial process by which stakeholders or organizations work together towards a common goal’, inter-organisational collaboration is synonymous with the ‘joint development of structures in which decisions are made, resources shared, and mutual authority and accountability exercised’ [2]. Such arrangements have a long and complex history in the National Health Service (NHS) in England since the 1960s [3]. Auschra (2018) documents how inter-organisational collaborations in healthcare can appear in several forms, as dyadic relationships between two partner organisations, or as inter-organisational networks [4]. Such collaboration can be defined as a cooperative relationship negotiated in an ongoing communicative process [5] or as a relationship that is mandated through government intervention [6]. In the UK, current emphasis is being placed on groups, networks, mergers, and buddying as solutions for resolving severe funding shortfalls as well as improving the quality of care provision between providers and regions. Such developments can be situated within a move towards Integrated Care Systems that is currently mandated in England and due to come into force in 2021 [6–8]. Furthermore, the response to COVID-19 has emerged as a timely example of unprecedented collaboration across organisations and sectors as the NHS responds to the pandemic [9, 10].

Inter-organisational collaborations can take many different shapes and forms. Whether associated with terms such as partnership working, partnering, or integration, these entities have been touted to bring a range of advantages over competitive approaches by enabling innovations [11], improving coordination of effort (i.e. reduce duplication, improved information sharing), enabling access to greater resource [12], gaining greater influence over others [13], and strengthening relationships [14]. Much of what has been learnt about these collaborative efforts finds such interventions to be extremely complex, often resulting in a myriad of unforeseen consequences. For example, conflict may arise over goals and objectives [12], organisations involved can suffer a ‘loss of glory’, loss of reputation and identity, or even performance losses that persist for long periods of time [1, 11]. As working collaboratively is so logistically difficult, and with possible pitfalls so severe, some claim that ‘it is generally best, if there is any choice, to avoid collaboration’ [15].

### Rationale for study

Notable contributions have sought to better understand how and where inter-organisational collaborations can work in healthcare contexts. However, to date, there remains limited understanding of how other inter-organisational entities such as strategic alliances, joint ventures, or buddying collaborations work, for whom, and in what circumstances. Although general theories of how inter-organisational collaborations work have been proposed, many questions remain about *how* interactions between cultures, as well as leadership, governance, financial, and other factors, can more specifically lead to strengthening or weakening of collaborations from a realist perspective [1, 15].

A realist perspective has the potential to enable a greater understanding of the mechanisms for *how* and *why* inter-organisational collaboration between healthcare provider entities can be achieved within complex adaptive systems [16]. Some authors have recently explored barriers to collaboration in integrated care [4], and others, the ‘success factors’ [17]. A variety of evidence reviews have also documented different perspectives regarding inter-organisational innovations [18], improvement initiatives of all types [19], and collaborations between healthcare services and higher education organisations [20]. One of the foremost examples of these analyses is by Turrini et al., who reviewed theoretical and evidence-based studies regarding determinants of network effectiveness [21]. These authors identified a range of contextual and structural elements, including network size and degrees of formalisation; however, their analysis stops short of uncovering how these contextual factors change the activation of the mechanisms driving collaboration itself. Use of a realist methodology would enable this greater understanding.

Synthesising learning from the successes and failures evident in previous evaluations of partnering is challenging. Collaborative arrangements vary by underlying drivers, how they are planned and implemented, and what they seek to achieve. In essence, they can be seen as a variety of interventions that are underpinned by a common thread of agreeing to move towards collaborating rather than competing. However, some are associated with the consequences of competition-orientated policies, such as a merger or acquisition of a ‘failing’ hospital. Another collaboration could be between voluntary participants to, for example, strengthen treatment pathways for a local population. Although there is recognition that the ways in which such collaboration is planned and implemented affect its success, in analyses of ‘barriers and facilitators’, the underlying drivers (that are frequently ‘political’ in nature) often remain unexamined [22]. Furthermore, while identifying barriers and facilitators is useful, this approach does not always recognise that different barriers interlock and create complex, system-wide challenges that cannot always be anticipated nor addressed by conceptualising and addressing barriers in isolation from each other [4, 23].

Inter-organisational collaborations can be seen as interventions that frequently fall foul of what Dixon-Woods and Martin term ‘magical thinking’; that is, it is assumed that ‘doing X’ will lead to outcome Y without any deeper logic behind how and why this change will occur [24]. This means that, often, the assumptions underlying how collaboration is intended to work are left implicit. Any collaborative effort is likely to have a long and complex implementation chain from initial discussions between stakeholders to the realisation of its intended benefits and is only as strong as its weakest link. Using a realist methodology to identify when, how, and in what circumstances these links break or hold, as well as why collaboration may lead to better performance, can support future quality of implementation. Finally, the evidence underlying inter-organisational collaboration is uneven in volume and quality and largely comprises local evaluations and grey literature. A realist methodology enables synthesis of all these literature types while acknowledging the complexity of the interventions that constitute inter-organisational collaborations in healthcare.

### Objectives

This review uses realist methods to draw on a range of evidence to formulate testable programme theories expressed in terms of explicit context-mechanism-outcome configurations (CMOCs) that explain how inter-organisational collaborations in healthcare work, to what extent they function, why, and in what circumstances.

In doing so, the review aims to lay out our initial configurations regarding how context shapes the mechanisms through which inter-organisational collaborations work across different boundaries, providing much needed understanding about how collaborative activities are associated with establishing and maintaining collaboration [8].

## Methods

### Rationale for, and use of, realist methods

Realist methods are built upon the epistemological approach of critical realism, which subscribes to the concept of generative causation [25]. Generative causation implies that mechanisms generate outcomes and that these mechanisms are context-sensitive [26]. In realist terms, contexts refer to the situations into which interventions are introduced that affect the operation of the intervention mechanisms [25]. An intervention may work through one mechanism in one set of contextual features, but work through a different mechanism, producing a different outcome, in another set. As a result, context and mechanism are keenly interlinked and cannot be separated [25]. Mechanisms, in realist terms, are the interactions between programme resources and the changes in reasoning by programme actors that occur as a result — both of which can be mechanisms in their own right [27]. Oftentimes, these mechanisms are not directly observable but nonetheless can be explanations of why particular outcomes come to be [25].

This paper represents phase 1 of our realist synthesis process [28]. Building on our initial rough theory of inter-organisational collaboration [29], the purpose of this phase of our synthesis is developing testable programme theories, comprised of explicit CMOCs. The formation of CMOCs requires hypotheses to be formulated about how contexts shape the mechanisms through which interventions work to produce outcomes [28]. Typical of this phase of realist synthesis is the use of a range of literature types to elucidate contexts, mechanisms, and outcomes, and we sought both peer-reviewed and grey literature across opinion pieces, commentaries, case studies, theoretical papers, and reviews to this aim [30]. After this phase, once our initial CMOCs are established, we can then, in the next phase, test our theory with further case studies, to give the theory refinements depending on collaboration type, location, and reasons for entering into collaboration across different contexts. Our synthesis will therefore be further developed with evidence generated from phase 2 testing and refinement of how and ‘for whom’ these partnering activities work in practice [26, 31]. This paper was written according to RAMESES reporting standards for realist syntheses (see Additional file 3) [32].

### Scoping of the literature

Scoping of the literature, or our pre-review phase of realist synthesis, has already been performed in another paper, which relied on a purposive search of academic and grey literature to identify an appropriate typology of collaborative arrangements and outline an initial rough theory of elements key to collaborations including governance, leadership, and culture. It also outlined initial propositions of how collaboration may work [29]. This informed the direction of this paper and aided in the design of the systematic searches. Additionally, a panel of expert advisory group members involved in organisational and policy-level decision-making with respect to collaborations in the UK’s NHS provided feedback on this initial phase of our realist synthesis.

### Searching processes

Searching processes in realist reviews tend to be evolutionary in nature, and that was the case here [32]. Initially, systematic searches were conducted to gather evidence around how inter-organisational collaboration works and what the contextual factors shaping success in a healthcare setting are, encompassing a wide range of entities such as alliances, buddying, mergers, acquisitions, and hospital groups. These searches were run between 20.02.20 and 04.03.20 on databases including the Healthcare Management Information Consortium (HMIC), MEDLINE, Social Policy and Practice, and PsycINFO (see Additional file 2 for search strategies). The HMIC commentary search (Additional file 2) was run on 12.01.2021 during resubmission of the manuscript. These searches were limited to 1990 onwards to provide the most up-to-date literature. Additionally, a Google Scholar search was conducted on 11.03.20 to identify any grey literature or papers missed. This search used the terms ‘theory organisational collaboration’ to identify theoretical papers and ‘inter-organisational collaboration healthcare’ to identify reviews and case studies. Reference scanning and citation tracking were also employed to ensure as many papers were identified as possible. Please see Additional file 2 for the full systematic search strategy.

After data synthesis, we realised that we lacked elucidation on some of the mechanisms underlying how leadership, amongst other elements, may be key to understanding the process of collaboration. As such, a non-systematic, purposive search was also used to identify middle-range theories (MRTs) which would allow us to gain further insight into mechanisms uncovered through our analysis of papers identified in our initial searches. These were identified using terms and combinations of terms such as ‘inter-organisational conflict’, ‘inter-organisational communication’, ‘inter-organizational trust’, ‘organisational capacity’, ‘collaborative leadership’, ‘organizational flexibility and effectiveness’, ‘collaborative accountability and governance’, and ‘collaborative regulatory environment’. These searches were conducted in Google Scholar in May 2020. Lastly, in response to reviewer comments on the originally submitted version of the manuscript, we conducted an additional Google Scholar search in December 2020 using the terms ‘confidence’ and ‘trust’, ‘formalisation’, ‘contract’, ‘contractualization’ combined with ‘inter-organisational collaboration’ or ‘partnership’ or ‘network’ for further MRT papers.

### Selection and appraisal of documents (relevance)

Selection of documents was performed on the basis of relevance to the realist synthesis, as is typical of a realist review [26]. The systematic review used the following inclusion criteria for the title and abstract stage: ‘the paper clearly relates to collaborations between one or more public sector organisations on either a structural or individual level’ and ‘the paper is a case study, evaluation, opinion, or review’. In the full-text screening as well as that for relevance, the paper had to include ‘propositions about the success or failure of collaboration in the public sector, mechanisms underlying how collaboration works, or include information about entry points (i.e. drivers of collaboration)’. Exclusion criteria for all stages included papers that ‘relate to collaborations or partnerships between staff and patients rather than between organisations’. Titles and abstracts were screened by JA with a subset of 10% screened by R Millar. Subsequently, papers were also excluded if they did not provide sufficient descriptive depth (relevance) to shed light on the workings of inter-organisational collaboration.

### Data extraction

Data extraction was carried out by one reviewer (JA), which involved combing the included papers for information relating to mechanisms underlying collaboration, programme theories, and contextual factors—often termed ‘success factors’ or barriers. As is typical of a realist review [28], identified passages in the documents were highlighted for relevance, before being extracted into separate documents according to realist logic and how they aided in understanding the intervention. This was performed using custom data extraction forms. These are available on request from the corresponding author.

### Synthesis and analysis process

The highlighted passages from the included documents were coded according to whether they shed light on entry points into partnering, contextual factors, and mechanisms. These could also include other elements relating to collaborations that helped elucidate the underlying ideas and assumptions regarding how partnering was intended to work and the sorts of contextual features that might shape the different mechanisms underpinning them. These were then extracted into a table similar to the one in Additional file 1. The majority of success factors and barriers were typically identified to be the inverse of one another, so these were amalgamated into becoming contextual factors at a later stage of the synthesis. As more papers were extracted, categories which were found to be thematically similar were merged to result in the final categories seen in this review. Contextual factors, mechanisms, outcomes, and entry points into collaboration were coded separately but contextual factors had their posited underlying mechanisms recorded alongside them, as well as any potential outcomes. The sources which supported the existence of these contextual factors were also recorded. Synthesis results were regularly discussed by JA and R Millar to maintain validity and consistency.

In some cases, mechanisms were explicit in papers identified in the systematic review, and in other cases, evidence was missing. As such, in cases where analysis was completed and mechanisms were missing, a purposive search was used to locate middle-range theories (MRTs) that could elucidate mechanisms which were triggered by these contextual features inherent to collaborations. Contextual factors were then clustered according to their underlying mechanisms and the case study and review literature, and MRT evidence synthesised. The theoretical clarity of mechanisms and the evidence underpinning them were discussed by two authors: JA and R Millar, and CMOCs were then formed. Included documents then underwent a second pass using specific search terms related to mechanisms and identified contextual factors to ensure all sources of relevant information were included.

We also sought to identify ‘entry points’ or drivers of collaborating and how these may shape the process of collaboration. However, during the process of data extraction for these entry points, we identified a lack of descriptive detail about how these entry points affect the mechanisms of collaborating. As such, thematic analysis was chosen over realist forms of analysis for this subcomponent of this review to simply outline and categorise which entry points we identified.

### Risk of bias and quality appraisal (rigour)

As is often the case with realist reviews, studies were not objectively assessed for quality against a checklist, as the studies included were of wide-ranging designs and we did not find it pertinent to the research question to exclude papers based on methodological quality [26]. However, as is the case with a typical realist review, studies were considered for their rigour while balancing their usefulness towards the realist synthesis. In line with guidance from Wong [33], the screening for rigour was ongoing during the analysis process and aimed primarily to increase the trustworthiness of the findings. For case studies and reviews, this process involved including a CMOC only when supported by (1) clear data in included studies and (2) by multiple sources [33]. For theoretical sources of evidence, only theories that had seen significant use in the literature since publication were used in the building of our MRT and CMOCs. If documents were screened out on the basis of trustworthiness, the reasons for doing so were to be recorded. However, no studies or extracts were excluded on this basis.

## Results

### Paper selection (systematic review)

**Fig. 1:**
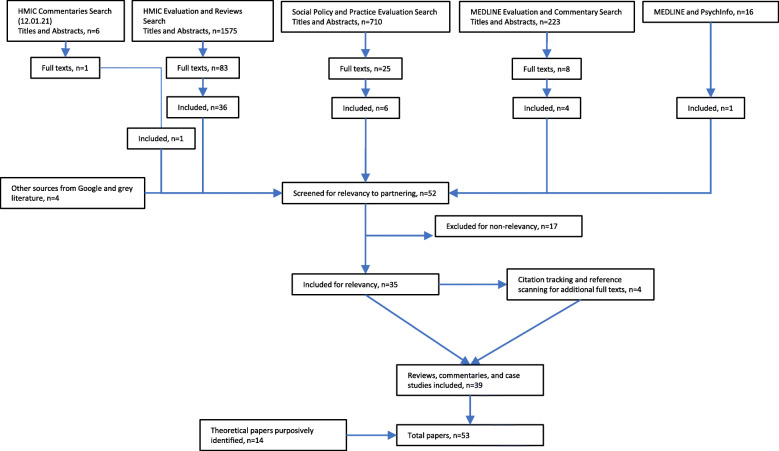
PRISMA flow diagram of paper selection from the systematic search

From the systematic search, a total of 2769 titles and abstracts were screened, which were filtered down to 117 full texts. The Google Scholar searches conducted on 11.03.20 produced 426,000 results on this specific day, and the first 40 pages of results were screened, resulting in four further papers. At this stage, 52 papers were included (Fig. 1). These were then screened for relevancy, i.e. whether these papers included sufficient descriptive depth regarding contextual factors, mechanisms, and outcomes underlying inter-organisational collaboration, which resulted in 35 included papers. Reference scanning and citation tracking resulted in a further four papers, giving a total of 39 papers included in this final analysis (Fig. 1) [1, 4, 12, 13, 15, 17, 22, 34–65]. Agreement between independent reviewers was 100%. Fourteen purposively identified papers were also drawn upon, which outlined MRTs, used to elucidate the workings of mechanisms, bringing the total number of papers included to 53.

### Document characteristics

Included studies comprised case studies (*n* = 18), reviews (*n* = 16), case-control studies (*n* = 2), surveys (*n* = 2), and theoretical papers (*n* = 1). Unfortunately, we were not able to identify any opinion pieces or commentaries that had sufficient analytical depth to inform the review. In terms of types of collaborations, included literature covered mixed collaborative types (*n* = 16), mergers (*n* = 9), alliances (*n* = 3), joint working (*n* = 2), contracting (*n* = 1), joint commissioning (*n* = 1), integrated care (*n* = 1), vanguard arrangement (*n* = 1), accountable care organisations (*n* = 1), community health partnerships (*n* = 1), buddying (*n* = 1), primary care partnerships (*n* = 1), and combined trusts (*n* = 1) (Table 1).

**Table 1:** Characteristics of included systematically reviewed studies

Paper	Collaboration type	Research type
Adedoyin et al. [57]	Mergers	Case study
Auschra [4]	Partnerships (mixed)	Review
Ball et al. [36]	Community health partnerships	Case study
Billings and De Weger [50]	Contracting	Review
Cameron et al. [54]	Joint working	Review
Casey [53]	Partnerships (mixed)	Review
Cereste et al. [48]	Mergers	Case study
Das-Thompson et al. [62]	Partnerships (mixed)	Case study
Dickinson and Glasby [1]	Partnerships (mixed)	Review
Dickinson et al. [63]	Mergers	Case study
Douglas [49]	Alliances	Review
Dowling et al. [45]	Partnerships (mixed)	Review
Evans and Killoran [56]	Partnerships (mixed)	Review
Ferrier and Valdmanis [43]	Mergers	Case-control study
Fulop et al. [22]	Mergers	Case study
Gannon-Leary et al. [37]	Partnerships (mixed)	Case study
Gaynor et al. [38]	Mergers	Case-control study
Glasby and Dickinson [39]	Partnerships (mixed)	Review
Hearld et al. [58]	Alliances	Survey
Hudson et al. [52]	Partnerships (mixed)	Review
Hunter and Perkins [61]	Partnerships (mixed)	Case study
Huxham [15]	Partnerships (mixed)	Theoretical
Idel [59]	Mergers	Case study
Kendall et al. [65]	Partnerships (mixed)	Review
Kershaw et al. [42]	Partnerships (mixed)	Case study
Leach et al. [34]	Buddying	Case study
Lewis [64]	Primary care partnerships	Case study
Lim [55]	Mergers	Survey
Mandell and Steelman [44]	Partnerships (mixed)	Review
Murray et al. [47]	Accountable care organisations	Case study
NHS Professionals [17]	Partnerships (mixed)	Review
Peck et al. [60]	Combined trusts	Case study
Round et al. [46]	Joint working	Case study
Shaw [51]	Mergers	Case study
Starling [40]	Vanguard	Case study
The King’s Fund [41]	Joint commissioning	Case study
What Works Scotland [35]	Partnerships (mixed)	Review
Wildridge et al. [13]	Partnerships (mixed)	Review
Zuckerman et al. [12]	Alliances	Review

Theoretical papers included one paper for partnership synergy [66], one for trust [67], two for conflict [68, 69], one for power [70], one for coordination [71], one for leadership [72], two for organisational flexibility [70, 73], one for task complexity [74], two for confidence and formalisation [75, 76], and two for proximity theory [77, 78].

### Main findings

#### Middle-range theory and mechanisms

**Fig. 2:**
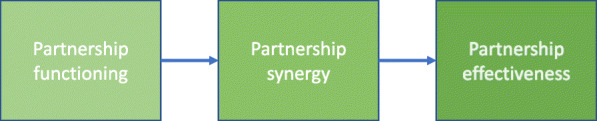
Partnership synergy as an intermediate outcome. Adapted from Lasker et al. [66]

Frequently mentioned in seven of the systematically reviewed studies was the concept of partnership synergy [4, 15, 36, 37, 39, 53, 63, 65], which was first coined by Lasker et al. [66] as a means for explaining *how* partnerships achieve advantage over independent, competitive working. As such, this theory was adopted as an MRT. This theory explains how there are ‘partnership functioning’ mechanisms essential to explaining the processes of working together, as well as ‘partnership performance’ mechanisms which underpin the improvements that collaborations seek to attain. Lasker et al. put forward partnership synergy as an intermediate outcome that comes after the functioning of the partnership, but precedes the effectiveness of it (Fig. 2) [66]. This means that when working well together, a combination of resources and skills of the partners is what enables achievement above and beyond what would have been possible individually. Partnership synergy can be considered a mechanism whereby a context of high partnership functioning leads to greater partnership synergy and thus improved partnership performance. Improved partnership performance is likely to be an outcome in itself, which results from a combination of sub-mechanisms, such as reduced duplication of effort, economies of scale, and competitive advantage [66]. However, these performance-related mechanisms are likely to depend on the aims and structure of each individual collaboration. We also add to the MRT the concept of collaborative inertia, which was put forward by Huxham [15] in one of the systematically identified studies. Collaborative inertia occurs when organisations and actors get ‘bogged down’ in the day-to-day functioning of the collaboration [15]. While trying to optimise the daily functioning of the collaboration, achievement of the actual aims of the collaboration fall by the wayside as significant manpower and time is devoted to partnership functioning rather than accomplishment of outcomes. It is possible that a collaboration will engage in a period of inertia in its earlier stages of formation, before synergy is later achieved. This concept of inertia was also put forward by a number of the included studies [15, 17, 52, 53, 63] and is defined as when ‘the output from collaborative arrangements often appears to be negligible or the rate of output to be extremely slow’ ( [13]; p. 403).

This MRT theory, taken together, proposes that the mechanisms comprising ‘partnership functioning’ need to have their context configured very favourably before synergy, and thus, enhanced performance, can be achieved. As partnership functioning relies on many other contextual factors and sub-mechanisms that enable collaboration, these will be explored in the following section.

#### Mechanisms underlying ‘partnership functioning’

The results of our analysis of the included studies identified a range of mechanisms underlying collaboration functioning, namely: conflict, trust, power, faith, interpersonal communication, leadership styles, cultural integration, and task complexity. We also identified a role for a ‘confidence’ in contract in certain circumstances. These mechanisms underline the ability for collaborations to perform through ‘partnership synergy’, and with avoidance of ‘collaborative inertia’. Although mechanisms can relate to both changes in reasoning and resources that an intervention introduces, the majority of mechanisms we identified relate to processes of reasoning by actors. This may be explained by our underlying assumption that ‘collaboration’ as an intervention in the inter-organisational setting is characterised by a change in organisational behaviour from competitive to collaborative behaviours [79]. The review also identified a range of contextual factors that affect how these mechanisms are activated (Additional file 1). The following sections present the interactions between these various elements.

##### Trust

Building and maintaining trust was a key mechanism identified by 16 papers in the review, and trust can be affected by a number of contexts [4, 12, 13, 15, 36, 37, 45–47, 52, 53, 57, 61]. As trust was mentioned so frequently, we sought to include an appropriate MRT for this element that explained how trust is linked to collaborative behaviour. Suitably, due to its use in many of the included studies (e.g. Axelsson and Axelsson [71]), we identified Vangen and Huxham [67] and their trust-building loop in inter-organisational collaborations as a suitable framework [31]. Trust has been defined in a myriad of heterogeneous ways, but we draw on the concept as a key component of social structures (organisations), with trust being formed as a result of networks and norms between actors in the social structure [80]. The trust-building loop proposes that a certain degree of trust is required to set the risk tolerance of each partner with respect to how ambitious the aims they agree to are. As more is accomplished by the collaboration, trust will be reinforced—but if failures occur, trust will be reduced; these successes or failures will again affect the risk tolerance in a cyclical manner [67]. This concept of risk tolerance allowed us to understand how greater trust enabled a greater tolerance for riskier endeavours, thereby changing bit by bit to what degree a partner would be willing to act collaboratively.

Trust underpins the majority of decision-making that is undertaken in a collaboration and also is tied keenly into other mechanisms such as respect, conflict, and power, which all may affect trust as an outcome [67]. For example, as previously mentioned, every time a conflict occurs between organisations, it is likely that trust between them will be reduced [69]. Trust is put forward by Vangen and Huxham to mean ‘the ability to form expectations about aims and partners’ future behaviours in relation to those aims’ [67]. Scholars argue that trust and risk are keenly interlinked, and trust is required to ‘take a risk’ in believing that a partner will do what is against their own interest for the collective good [81]. This places the ‘trustor’ in a vulnerable position relative to the trustee—and in most voluntary collaborations this goes both ways. When results of these risks arise, they can build more trust or have it broken down depending on the outcome. A number of contexts are important for modulating the initial level of trust with which partners enter an arrangement as well—which can act as a buffer against future conflicts and task failures.

*Trust building, synergy, and perception of progress*

**Fig. 3:**
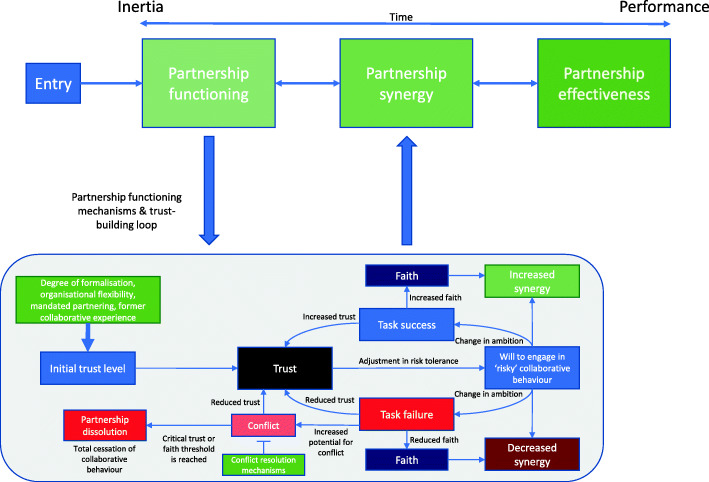
Programme theory—depiction of main mechanisms and outcomes at play

Trust building is another factor that needs to take place throughout the process of collaboration and is likely to be cyclical in nature, as acts which beget trust are usually reciprocated [67]. Mutual successes such as achievement of outcomes reinforce trust in both parties (Fig. 3) [67, 82]. This loop was explicitly mentioned by included systematically identified studies [13, 15, 53]. This means that outcomes need to be realistic and agreed upon by both parties; thus, if outcomes are too overambitious, then trust will also be reduced as they are unachievable (Fig. 3). Likewise, this links into the mechanism ‘perception of progress’, which is defined here as how well organisational actors perceive the organisation to be progressing towards the aims of the collaboration itself. Perception of progress as a mechanism links into both trust and faith as outcomes and is affected by a number of contexts outlined below.

This CMOC was supported by quotes such as the following by Round et al. (p. 300): ‘Challenges included a feeling that the programme had, ‘massively overambitious proposals in the original business case’ and was ‘too ambitious with a lack of realism’. This hampered progress to deliver the initial objectives…’ [46] as well as by Dickinson and Glasby (p. 819): ‘the tendency to see partnership working as a panacea to a series of current problems, placing too much faith in its ability to deliver a series of over-ambitious aspirations, therefore running the risk of disillusioning staff if such aspirations are not achieved; and undermining the subsequent partnership by failing to attend to practical details’ [1]. Relatively unambitious intermediate aims and outcomes formulated at the beginning of a collaboration may thus serve to solidify and build trust early on, enabling achievement of higher ambition ultimate outcomes, such as an improvement in care quality [83]. This means that:

*Unambitious aims (context) ➔ better perception of progress (mechanism) ➔ increased trust and risk appetite (outcome).*

As such, in some cases:

*Overambitious aims (context) ➔ reduced perception of progress (mechanism) ➔ conflict (outcome)* (Fig. 3).

Trust is also essential to maximising collaborative synergy. As Jagosh et al. and Lucero et al. have identified in their analyses of public sector partnerships, without trust, partners will not be able to work together in a functional manner [84, 85]. This means that:

*High trust (context) ➔ partnership synergy (mechanism) ➔ collaborative performance (outcome).*

*Historical context*

It is evident that a certain minimum level of trust needs to be maintained at all times for a collaboration to avoid dissolution, and that certain factors are likely to modulate the level of trust already in place when people begin to initially work together. As mentioned by papers in our systematic review, these factors could include whether the organisations involved have had pre-existing collaborations that were successful (or not) [13, 44, 52, 56], as well as the historical context of collaborations in the geographical area in which the organisations are located, and a partner’s reputation [4]. These factors have the potential to act as enablers or barriers to potential collaboration by modulating the pre-existing level of trust and suspicion with which partners will begin collaborating. This was supported by quotes such as the following from Auschra (p. 7): ‘if they have gathered experiences from former collaborations, organisations assess cooperation outcomes differently’ [4]. Thus, the following CMOC emerges:

*Existing successful collaborations (context) ➔ better initial trust (mechanism) ➔ greater ambition in objectives (outcome)* (Fig. 3).

*Formalisation*

The degree to which a collaboration is formalised was mentioned in included studies as a method of instilling trust between partners by cementing tasks and accountability in contractual, legal terms based on relational contracting [44, 53]. Formal agreements forged at the beginning of such arrangements in the connecting stage of collaboration can also serve as a scaffolding which holds up and solidifies trust between partners (Fig. 3) [82, 86]. This is because, as rules are laid down with a legal mandate to uphold them, there is an understanding that the other side will follow them. This is supported by quotes such as the following by Casey ( [53]; p. 78): ‘the more formalized a partnership is, the more likely it is to be maintained, because formal arrangements tend to signal commitment and accountability’ [47]. Thus, we hypothesise that:

*Legal agreements (context) ➔ greater initial trust (mechanism) ➔ greater risk threshold and perception of progress (outcome).*

The potential impact of formalisation on trust and confidence, depending on the collaboration type, is explored further in the discussion.

##### Conflict

*Inter-organisational conflict* A further mechanism explicitly mentioned throughout the included papers was conflict; many factors lead to conflict if not properly managed, including cultural differences, the management of individualist vs. collectivist interests, power dynamics, congruence of aims and objectives, whether collaborations are dissolved as appropriate, ongoing evaluation, organisational ownership of decision-making, and the pace of collaboration development [4, 13, 15, 46, 47, 55]. Ideally, all of these factors are overseen by conflict resolution mechanisms that rely upon mutually agreed governance and accountability arrangements between partners. However, there are also other mechanisms at play that can prevent conflicts arising before they even happen. For example, developing cultural integration plans which ensure that conflicts arising due to cultural differences in workforces are planned for and mitigated [60].

As mentioned by Lumineau et al. [69], conflict between organisations is often very different from interpersonal conflicts due to the level of interaction, decision-making parties, incentives and motivations of key stakeholders, governance structures for preventing and managing conflict, repair mechanisms available for resolution of disputes, and the institutional context. Essentially, the situations become much more complex due to the myriad actors and mechanisms involved. Conflicts can also take numerous forms, such as whether they are competence-based (relating to skills or knowledge of partner) or more fundamental, integrity-based conflicts [69]. These have differing implications for how resolvable they are with different management strategies.

Perhaps the most pertinent categories of management strategies include constructive (joint problem solving and persuasion) vs. destructive (domination) conflict resolution strategies, which are evocative of the type of relationship that is at play between partners [68]. The outcomes of conflict are typically a function of the effectiveness of the conflict resolutions in place and the type of relationship which already existed [69]. As already mentioned, this could manifest in a loss of trust, or, in the case of re-commitment that arises from a constructive management process, even improved trust due to a gain in collaborative working spirit. We suggest that conflict is keenly linked to trust and that conflict can be both a context and a mechanism depending on the element of analysis (Fig. 3). For example:

Included papers (e.g. Murray et al. [47]) suggest that:

*Conflict between partners (context)* ➔ *can lead to reduced trust (mechanism)* ➔ *reduced ambition and faith in the collaboration (outcome)*

Likewise, others (e.g. Auschra [4]) suggest that:

*Having a shared vision (context) ➔ is likely to reduce conflict (mechanism) ➔ leading to improved trust (outcome)*

*Conflict (context) ➔ constructive conflict resolution strategy (mechanism) ➔ lowered reduction to trust (outcome)*

*Conflict (context) ➔ destructive conflict resolution strategy (mechanism) ➔ reduction to trust (outcome)*

*Accountability and commitment*

As discussed previously, accountability and conflict resolution mechanisms are key, and should be established as a part of the governance of the arrangement in the planning phase of a collaboration [82]. Effective conflict resolution and accountability processes are essential to modulating the impact that conflict has on the collaboration itself, as mentioned by studies in the systematic review [47, 53, 65]. Conflict causes loss of trust, loss of faith in the collaboration, and loss of perception of progress, but, if effective measures are in place, then the likelihood of conflict spiralling out of control and causing the downfall of the partnership is much lessened. This potential for conflict causing dissolution and loss of trust is supported by quotes such as the following from Murray et al. (p. 775): ‘For Access ACO (Accountable Care Organisation), these tensions were resolved through active conflict resolution, and the alliance remained intact throughout our research period. In contrast, for Collaborative ACO growing distrust paired with the management partner’s decreased investment and high fees prompted dissolution’ [47]. Likewise, the inverse is true as well [69]. As such:

*Preparedness for conflict and accountability (context) ➔ reduced conflict (mechanism) ➔ a smaller reduction in trust (outcome)* (Fig. 3).

*Intra-organisational conflict*

The literature reported that conflicts can also arise within organisations involved in a collaboration, which have the potential to reduce the organisation’s effectiveness [12, 54, 59]. For example, workforce churn brought about by people leaving the organisation, due to the additional workload brought about by the partnership, or due to other factors such as pay imbalances, will likely lead to conflict within an organisation and reduce organisational effectiveness [53, 61]. Likewise, if lower-level staff are not involved in the decision-making around collaborative involvement and the shape the entity should take (context), then there may be conflict (mechanism), which could lead to a reduction in faith (outcome) [12]. Rather than in the case of inter-organisational conflicts, if managed prior to getting out of control, conflicts within organisations can likely be dealt with without involving the other partner. Factors such as workforce churn could easily be noticed by another partner and have potential to lead to conflict. However, if managed properly, it should not escalate to such a degree. Intra-organisational conflict reduces the ability for an organisation to accomplish the aims of a partnership by wasting organisational time on conflict resolution and by reducing faith in the partnership. In this case:

*Intra-organisational conflict (context) ➔ reduced perception of progress(mechanism) ➔ reduced faith (outcome).*

##### Power

Power was mentioned throughout the included papers as a key mechanism underlying collaborative efforts [4, 13, 17, 47, 61]. Power refers generally to the influence one organisation has over another. Power can stem from hierarchical position, control over critical or scarce resources, and from discursive legitimacy, or ability to mobilise external support [87]. Power relations are also key to trust building; some arrangements can be characterised by a dominant partner controlling the agenda to protect its own interests. This is supported by quotes such as the following by Murray et al. (p. 767): ‘A power-sharing approach and consistent investment in the community with support for local ACO-level decision making fostered trust at the leadership level between the ACO and the management partner’ [47], In lopsided relationships in terms of organisational size, the larger one may dominate [67]. This has the potential to skew the trust relationship by lowering the initial degree of trust. In cases where collaboration is enforced by a governmental organisation, such as with buddying or competition-related acquisitions in the UK’s NHS, these power dynamics may be intrinsic to the relationship and therefore it may be very difficult to build trust [8, 88]. Willem and Lucidarme [70], in their review and test of the role of trust in inter-organisational networks, propose that mandatory networks are likely to be less effective and have reduced levels of trust.

*Lopsided power in relationship (context) ➔ domination by one partner (mechanism) ➔ reduced trust (outcome)*

*Reduced trust (context) ➔ reduced risk threshold and aim ambition (mechanism) ➔ reduced achievement (outcome)*

##### Resource use

Douglas [49] posits that resource exchange during partnerships relies heavily on the power dynamics within the relationship. A more dominant partner may take more resources for themselves, or dependencies may develop whereby the ‘weaker’ partner is dependent upon the stronger one for resource, which could be called an ‘unhealthy’ power dynamic [47]. This ‘unhealthy’ power dynamic in which one partner dominates is characterised by any scenario in which another partner has a loss of trust arising from the dynamic. In one of the cases of a healthcare alliance analysed by Murray et al., cost savings that were garnered by the alliance were sequestered by the management partner, who was dominating in the power structure, fatally reducing trust and causing the end of the alliance [47].

*Unequal resource distribution ➔ domination in power hierarchy (mechanism) ➔ reduced trust*

##### Faith

Related to trust is the concept of faith, which may also be expressed as confidence or belief in the collaboration itself. While trust always relates to inter-organisational relations and belief in one’s partner, faith relates more to how actors within one or more organisations continue to believe in the collaborative endeavour as something of value. While trust is likely to modulate faith to a certain degree, as low trust in a partner could affect faith in the partnership, a scenario is also foreseeable where a partner has low trust but high faith. One partner could have been let down repeatedly by another partner in the achievement of the aims of the partnership, leading to low trust. However, this partner may not yet be ready to give up on the concept of collaboration itself, as the plan is strong and the logic for how collaboration can achieve the intended outcomes for stakeholders is still clear. The concept of faith was not explicitly articulated in any of the included studies, but some have highlighted the importance of confidence and belief in the collaborative arrangement, which is a roughly analogous concept [37, 47, 48]. We posit that faith/confidence/belief is a distinct mechanism from trust that, based on included studies, is chiefly affected by two contextual factors: ambition and authenticity of the collaboration. These can serve to modulate the faith which actors hold in the collaboration. It is possible that there is a also faith-building loop that exists within each involved organisation in a collaboration, similarly to the trust-building loop. In this sense, faith is also essential to collaborative synergy as it upholds collective desire to work on collective goals. As such:

*High faith (context) ➔ partnership synergy (mechanism) ➔ collaborative performance (outcome)*

*Low authenticity of the collaboration (context) ➔ low faith in arrangement (mechanism) ➔ lower partnership synergy (outcome)*

*Ambition and authenticity*

We suggest that ambition is to what degree the aims and outcomes set in the planning phase of the collaboration are realistic (and feasible) [1, 37, 46, 52]. The degree of ambition needs to be kept realistic to ensure that there is a perception of progress and the building of trust between partners, as well as to maintain faith in the relationship. As Round et al. [46], p. 300 mention in their case study of an integrated care programme, the initial plan was ‘too ambitious with a lack of realism’, and this ‘hampered progress to deliver the initial objectives’.

*Ambition is too high (context) ➔ reduced aim achievement (mechanism) ➔ reduced faith and trust (outcome)*

Authenticity is another consideration, and it refers to whether the collaboration actually is based upon a real need to solve a problem, or whether a collaboration is simply undertaken to look ‘trendy’ and virtuous [1, 61]. Inauthentic collaborations are unlikely to inspire workers to put significant effort into collaborating.

*Inauthentic collaboration (context) ➔ reduced faith in collaboration (mechanism) ➔ reduced aim achievement (outcome)*

##### Coordination

Increased coordination is often one of the primary motivations for organisations seeking to cooperate. Coordination refers to a reduction in duplication of effort, reduction in gaps of services, and sharing of knowledge and skills [4, 52, 71]. The degree to which organisations are coordinated is a key mechanism underlying success and failure of collaborations. Coordination is a mechanism that is linked to mechanisms of information exchange and interpersonal communication.

*Information exchange*

A common concern in the literature is the ability to exchange information between partners as required, which is often key to properly coordinating delivery of care and other aspects of work [4, 17, 54, 57, 61, 89]. A lack of information exchange leads to a lack of coordination:

*If information is not exchanged as required (context) ➔ a lack of coordination can occur (mechanism) ➔ leading to conflict (outcome)* [4, 54].

Key to the sharing of information is the interoperability of, and devotion of resources to, information technology systems [17].

*If more interoperable systems are already in place (context), ➔ a reduction in task complexity (mechanism) ➔ will make combining these systems more straightforward, improving perception of progress (outcome).*

*Interpersonal communication*

Dialogue between actors is required in order to build the trust required by the collaboration, transfer information, and properly coordinate tasks [4, 53, 61]. However, just as communication is required to build up trust, so too is trust required for actors to desire to communicate [53]. The essential role of communication in building trust is supported by excerpts such as the following by Wildridge et al. (p. 7): ‘The role of clear, consistent communication (in trust-building) is at least implicit and sometimes explicit in much of the literature’ [13]. As such:

*Greater interpersonal communication (context) ➔ increased trust (mechanism) ➔ increased synergy (outcome)*

However, this element can go both ways and depends upon the culture of the actors interacting.

*If there are conflicting cultures between partners (context) ➔ then increased interpersonal communication (mechanism) ➔ may lead to conflict (outcome)*

Likewise, the inverse is also possible (i.e. that more related cultures lead to reduced conflict).

Interpersonal communication is also essential for collaborative synergy to be achieved and, thus, for performance to be maximised. As Lasker et al. (p. 194) put forward, ‘Effective communication strategies and mechanisms to coordinate partners’ activities are needed to facilitate synergistic thinking and action’ [66]. Thus:

*Increased interpersonal communication (context) ➔ partnership synergy (mechanism) ➔ increased collaborative performance (outcome)*

*Stakeholder involvement*

According to the included papers, communication with and involvement of stakeholders can make the difference between a collaboration being taken seriously or not [13, 36, 40, 48]. Inclusion of these stakeholder perspectives allows for definition of the correct priorities and focusing on delivering benefits where they are needed most. We suggest that the ‘engagement of stakeholders’ context is keenly linked into authenticity of the partnership, increasing faith, congruence of aims/objectives, and focus on the right outcomes. This is supported by excerpts such as the following from Wildridge et al. (p .7): ‘Inclusion of service users’ perspectives, for instance, can make the difference between a project being taken seriously or not’ [13]. The following CMOC is a result:

*Involvement of stakeholders (context) ➔ increased authenticity of the collaboration (mechanism) ➔ increased faith in the collaboration (outcome)*

##### Leadership

Eight of the included studies mentioned leadership as being key to the success of partnerships; however, few elucidated upon why [13, 34, 36, 47, 49, 56, 58, 61]. Evans and Killoran [56], in their realistic evaluation of five different models of partnership working, mention ‘leadership and management skills’ being enhanced through ‘external consultancy support’ and ‘strong project leadership’, but do not outline the mechanisms underlying these aspects. Wildridge et al. [13] mention ‘continuing, visible, and joint commitment from individuals in positions of leadership and influence’ as very important, and Leach et al. [34] mention in their evaluation of a buddying programme that ‘compassionate leadership’ was key. Leadership is essential to any kind of organisation, regardless of whether they are collaborating with another, but is not in all cases a mechanism that underlies partnership, rather, can perhaps also be a context that frames the partnership from the outset. Nonetheless, it is possible that collaboration can introduce a change in leadership style towards one that may maintain a collaborative endeavour better than others. In this case, it would become a mechanism in a realist sense. Due to the lack of description of leadership mechanisms, to identify them, we turned to the theoretical literature.

According to Fillingham and Weir [72], in their study of integrated care leadership in the UK, leadership during partnership requires different skills than those displayed during their climb of the organisational ladder. Successfully collaborating requires the use of individual skills rather than their position, an ability to compete in a way that enhances the competition (through collaboration), conducting business ethically in a way that builds trust, and development of a process focus emphasising the ‘how’ rather than the ‘what’ [72]. Likewise, Hunter and Perkins [61] broadly agree, emphasising a participative and open leadership style of listening, asking questions, and co-producing solutions—a less dictatorial style [90]. Huxham [15] significantly expands on this by analysing the activities which leaders should be focusing on to move a collaboration forward, namely, facilitative behaviours which serve to involve and mobilise members. Huxham [15] also identifies a more combative leadership approach, which may occur in collaborations that have unequal power dynamics (such as those which are mandated), in which the leader engages in ‘collaborative thuggery’ to push out those who do not align with their vision of collaboration. We posit that this, too, may work in cases where trust was compromised from the outset and collaboration is mandatory in the first place, but would be likely to undermine trust and respect in voluntary collaborations. Depending on the situation, leadership may act as either a context for another mechanism, or a mechanism in itself, which allows collaboration to flourish or flounder (Table 3).

*Collaborative leadership style (listening, asking questions, co-producing solutions) (context) ➔ improved trust (mechanism) ➔ better aim achievement (outcome)*

*Mandated collaboration and very low trust (context) ➔ combative leadership approach, pushing out those who do not agree (mechanism) ➔ shared vision for partnership (outcome)*

*Voluntary collaboration (context) ➔ combative leadership approach, dominating power hierarchy (mechanism) ➔ reduced trust (outcome)*

Additionally, others have connected leadership keenly to the concept of developing and integrating culture, which is key to establishing greater trust and respect between workforces and leaders [91]. Factors relating to leadership which affected successful creation of a new culture included establishing organisation-wide communication channels and outlining outcomes for different staff types, involving more willing partners first, and leading in a positive and constructive manner [91].

*Positive leadership style (context) ➔ easier integration of cultures (mechanism) ➔ greater trust (outcome)*

While there are no doubt further, more detailed behaviours key to leadership styles that will uphold collaborations, these will be explored in greater depth in the next phase of theory refinement.

*Culture*

Culture, defined here as the attitudes and beliefs held by a workforce, is often cited as a primary reason for dissolution of collaborations, if conflicts arising from differences in culture are not properly mitigated [4, 17, 37, 46, 47, 51, 54]. Included studies suggest that auditing all organisations’ cultures, performing training around values and behaviours, implementing ongoing measurement, and even hiring and firing based on values may all contribute to general ‘preparedness’ to avoid conflict [47, 53, 65]. Thus:

*Preparedness for conflict through cultural similarities (context) ➔ reduce conflict (mechanism) ➔ reducing the impact on trust (outcome).*

Likewise, the distance in culture (which could be measured if one desired) is also likely to modulate the ease of integration in this respect. Thus:

*Cultural closeness (context) ➔ reduce conflict (mechanism) ➔ avoiding degradation in trust (outcome).*

Of course, as trust gradually reduces, it may reach a threshold at which dissolution of the collaboration occurs, and collaborators recede to competitive behaviour.

*Organisational flexibility*

According to Kopanaki and Smithson [73, 92], organisational flexibility refers to an organisation’s capability to face environmental disturbances, or adapt when confronted with new circumstances. Of course, flexibility will be a trait inherent to the organisation before it even enters the collaboration, which will modulate the ability, and speed, with which an organisation can pivot to working together collaboratively. As such, we have considered it a context rather than a mechanism per say. Flexibility and/or capacity were mentioned frequently in the included studies of the systematic review [13, 35, 52, 54, 56, 65, 89]. According to What Works Scotland (p. 8), ‘one of the most striking themes emerging from analysis of this results chain is the need for effective partnerships to develop clear structures and processes whilst allowing for flexibility, engagement and responsiveness’ [35]. Willem and Lucidarme [70] put forward the idea that collaborations are oftentimes intended to be more flexible alternatives to the status quo and that low flexibility can lead to an overly bureaucratic process, reducing trust. We suggest that flexibility is not a mechanism, but rather, a contextual element that impacts how well collaboration can be implemented. However, flexibility may be able to be enhanced through other means, and those means may be mechanisms in themselves. Therefore, we posit that:

*Greater organisational flexibility (context) ➔ increased trust due to improved goal achievement (mechanism) ➔ reduced conflict (outcome)* (Table 3)

##### Perception of task complexity

Perception of task complexity is a mechanism that underlines how actors perceive how difficult tasks are to complete, with more complex tasks requiring both more resources and more manpower to achieve [74]. Complexity as a mechanism was referred to by papers such as Kendall et al., which refer to ‘diversity and complexity of the problem’ as a key factor influencing success of collaborations; likewise, Mandell and Steelman refer to the ‘complexity of purpose’ underlying how difficult the aims of the collaboration are to achieve [44, 65]. This perception of difficulty is likely to feed into faith (i.e. the belief in the collaborative endeavour). Thus:

*Perception of great task complexity (context) ➔ lower faith (mechanism) ➔ reduced synergy (outcome)*

Organisational size is a key factor that the included studies proposed affects the initial complexity of the collaborative arrangement [38, 93], as well as the size or type of the problem that collaboration is intended to solve [44]. Gaynor et al. [38] used econometric modelling to assess the characteristics and impact of 102 acute hospital mergers that took place in the NHS between 1997 and 2002. They found that compared to matched control hospitals, mergers tended to involve smaller hospitals with weaker financial performance. The main impact of mergers over the subsequent 4 years was a reduction in capacity and associated activity, with comparatively little impact on a range of performance measures, and there was little evidence of an effect of size on merger success. Fulop et al. [22] also performed a qualitative study of mergers and found that increased size provided benefits in terms of having a larger pool of professional staff, increased attention from local authorities, and better cross-fertilisation of ideas. However, increased size also led to more remote senior managers, not enough cohesion through multiple levels of the workforce hierarchy, and a loss of informality and autonomy felt by those moving from smaller to bigger organisations. They found that:

*Merger of larger organisations (context) ➔ more task complexity (mechanism) and ➔ slowed decision-making and internal communication (outcome).*

As such, the organisational size is a context that primarily modifies the mechanism of task complexity, and as discussed above, achievement of more ambitious tasks requires greater risk threshold, meaning a requirement for greater trust [67].

*Larger organisational size (context) ➔ greater complexity of task (mechanism) ➔ greater trust requirement between partners (outcome)*

*Ease of acquiring partner*

On the whole, inter-organisational collaborations are entered into to solve a problem through some achievement that collaboration enables. Mandell and Steelman mentioned that collaborations founded upon solving simpler problems may make it easier to get potential partners to the table, as they may feel the stakes are low enough to ensure their individual goals can still be maintained and the expected difficulty of the task is reduced [44]. Simpler problems may also link into trust, as easier achievement early on in the process can reciprocally foster more trust between partners (Fig. 3).

*Simpler problem to solve (context) ➔ reduced complexity of task (mechanism) ➔ lesser trust requirement between partners (outcome)*

*Regulatory environment*

Regulatory bodies, i.e. those above the collaborating ones in the hierarchy (i.e. governmental or health regulation authority bodies), can impose legal or resource constraints, or incentives, for collaboration to occur. This was cited frequently as both a barrier and enabler for collaboration [4, 44, 46]. For example, in the UK, the Competition and Markets Authority has posed a barrier to circumvent for NHS providers seeking to collaborate by mandating a certain degree of competition to be in place [8]. Auschra [4] provides evidence that these barriers can manifest by forbidding collaboration entirely, stopping it before it even begins, or causing additional time and financial cost considerations (i.e. legal problems hampering information exchange, pooling of budgets, and bureaucracy requirements). For example, this is evidenced in this reflection by Das-Thompson et al. ( [62]; p. 26) regarding moving towards system-level collaboration in England: ‘current regulation of individual providers is acting as a barrier to integration, with limited incentives to encourage wider performance implications at system level’. Therefore, it is possible for a:

*Favourable regulatory environment (context) ➔ reduce task complexity (mechanism) ➔ enhancing perception of progress (outcome).*

This will go on to improve trust and faith. Vice versa is also possible, i.e. that an unfavourable regulatory environment increases task complexity, due to its presence as a barrier and facilitator in the literature.

*Geographical proximity*

Geographical proximity of partners has been lauded as a contextual element which can lead to failure if not considered highly enough in the initial phases of a collaboration [4, 42, 46, 54]. Geographical co-location enhances casual face-to-face interactions (both planned and impromptu) and can be responsible for ‘encouraging informal contact, which increased mutual understanding’ ( [68]; p. 230). Geographical proximity therefore fosters information transfer and trust through improved mutual understanding [77, 78]. However, some outline that it may also lead to greater informality which has potential to undermine any perception of progress [54]. As such:

*Greater geographical proximity (context) ➔ enhanced interpersonal communication (mechanism) ➔ fostering trust (outcome).*

#### Point of entry into collaborating

The point of entry into a collaboration is highly likely to shape the nature of the relationship that follows [14, 92]. In addition to the contextual factors in Additional file 1, our review sought to identify entry points and drivers for partnering in included studies and categorised them using inductive thematic analysis into 6 categories (Table 2). In the case of seeking to partner due to funding and resource concerns, market opportunities, or innovation, a collaboration is likely to be shaped primarily by the regulatory environment, history of prior collaboration, and stereotypes from knowledge of other collaborations in the area. All of these factors together serve to form the context to determine the initial level of trust.

**Table 2:** Coding of entry points into collaborations

Number	Entry point	Explanation and sub-elements	Source(s)
1	Funding, resource, efficiency concerns	Need to partner to avoid financial or resource shortfall	Cereste et al. [48]; Dickinson and Glasby [1]; Fulop [93]; Gaynor et al. [38]
2	Existing interdependence	A natural evolution of existing collaborative arrangements	Douglas [49]; What Works Scotland [35]
3	Threats from competitors or markets	Partnering to avoid loss of competitiveness/autonomy	Murray et al. [47]
4	Market opportunities	Partnering to secure an advantage in the marketplace	Murray et al. [47]
5	Policy directives	Mandated partnering from governmental sources, e.g. buddying from special measures, forced acquisition/merger	Fulop et al. [22]; What Works Scotland [35]; Gaynor et al. [38]; Lim [55]; Hunter and Perkins [61]
6	Innovation	Desire to partner with a specific goal in mind that cannot be achieved in current organisational form	Hudson et al. [52]; Hunter and Perkins [61]

In the case of existing interdependence, it is likely that it will be a collaboration moving from a less integrated form to a more integrated form, i.e. they may have been members of a network but are now moving to a full merger. In this case, further collaboration is unlikely to be considered unless they already have a positive relationship; thus, the initial level of trust is likely to be elevated. As mentioned previously, policy directed (i.e. mandated) partnering are likely to drastically lower trust and power dynamics from the outset. This is supported by Auschra (p. 7), who states ‘if a (mandated) collaboration threatens the political and economic interests of an organisation involved, it can be very reluctant to collaborate’ [4]. Likewise, entering a collaboration primarily with the goal of avoiding marketplace threats is also likely to lower trust, as an organisation will still be entering the arrangement from a standpoint of relative negative performance and fear.

The role of entry points and drivers will be fully explored in a future paper as another part of our realist synthesis, due to length considerations and the requirement for further systematic searching. However, in this current paper, we posit that:

*Mandated collaboration (context) ➔ creates power imbalances (mechanism) ➔ reduced initial trust (outcome).*

Mandated collaboration is likely to require significant early ‘wins’ to enhance perception of progress, and mutual respect/goodwill to overcome. This will be explored further in the discussion.

## Discussion

### Explaining the programme theory

Table 3 depicts the CMOCs that have been identified in the included articles, which represent the essential mechanisms at play and the contexts which affect them. In summary, these CMOC arrangements propose that, for inter-organisational collaborations to function, trust and its link to risk tolerance, as well as faith, are essential to driving  collaborative behaviour. Similarly, when too many conflicts have occurred, and too many tasks have not been achieved, trust in the collaboration can be broken, leading to dissolution. Many contextual factors, most of which are similar to those identified by prior literature [21], such as former collaborative experience, serve to modulate the initial trust and faith levels when entering the collaboration.

**Table 3:** Summary of proposed CMOCs

Context	Mechanism	Outcome(s)
**PARTNERSHIP PERFORMANCE**		
**Synergy and collaborative inertia**		
High partnership functioning and trust level	Synergistic working	Greater task achievement
Low partnership functioning and trust level	Collaborative inertia	Lesser task achievement
High faith	Synergistic working	Greater performance
Greater, appropriate strategies of interpersonal communication	Synergistic working	Greater performance
Greater confidence (in mandated or integrative collaboration)	Synergistic working	Greater performance
**Perception of progress and performance**		
Intra-organisational conflict	Lessened perception of progress	Reduced faith
Unambitious aims	Lessened perception of progress	Reduced faith
Absent actors	Lessened perception of progress	Reduced faith
Greater organisational flexibility	Enhanced perception of progress	Enhanced faith
Workforce instability	Lessened perception of progress	Reduced faith
Conducting ongoing evaluation	Enhanced perception of progress	Increased faith
Clarity of roles	Enhanced perception of progress	Enhanced faith
**PARTNERSHIP FUNCTIONING**		
**Conflict**		
Appropriate accountability arrangements	Reduced conflict	Enhanced trust
Slow pace of development	Conflict	Reduced trust
Higher cultural compatibility	Reduced conflict	Enhanced trust
Shared vision	Reduced conflict	Enhanced trust
Improper cessation of collaboration	Increased conflict	Reduced trust
Task failure	Increased conflict	Reduced trust
**Trust**		
Destructive conflict resolution strategy	Reduced trust	Reduced faith in partnership, reduced perception of progress
Constructive conflict resolution strategy	Improved trust	Improved faith in partnership improved perception pf progress
Existing successful collaborations	Better initial trust	Greater ambition in objectives
Legal agreements	Greater initial trust	Greater risk threshold, greater ambition
Reduced trust	Reduced risk threshold and aim ambition	Reduced perception of progress
Overambition	Lack of achievement	Reduced trust
Underambition	Lack of achievement	Reduced trust/faith
Significant conflict	Critical trust threshold reached	Partnership dissolution
**Confidence**		
Greater formalisation (in context of mandated or integrative collaboration)	Greater confidence	Increased synergy
**Power**		
Larger organisational size	Domination by single partner	Reduced trust
Unequal resource distribution	Domination by single partner	Reduced trust
Mandated collaboration	Power imbalance	Reduced initial trust
**Faith**		
Involvement of stakeholders	Increased authenticity of partnership	Increased faith
Inauthentic partnership	Reduced faith in partnership	Reduced perception of progress
Reduced faith	Critical threshold of faith reached	Partnership dissolution
**Interpersonal communication/coordination**		
Conflicting cultures	Increased interpersonal communication	Conflict
Greater geographical proximity of partners	Increased interpersonal communication	Fostering trust
**Leadership**		
Collaborative leadership style	Improved trust	Better perception of progress
Mandated collaboration characterised by low initial trust	Combative leadership, pushing out of dissenting partners	Shared vision
Voluntary partnership	Combative leadership approach	Dominating power hierarchy
**Cultural integration**		
Inclusive leadership style	Easier cultural integration	Greater trust
**Perception of task complexity**		
Interoperable systems	Reduction in perception of task complexity	Enhanced interpersonal communication
Larger organisational size	Greater perception of task complexity	Greater requirement for initial trust due to greater perceived risk
Larger organisational size	Greater perception of task complexity	Slowed communication
Simpler aims of collaboration	Reduced perception of task complexity	Lesser trust requirement
Favourable regulatory environment	Reduced perception of task complexity	Enhanced perception of progress

A sense of faith in the collaboration itself must be maintained, which is intricately linked to the trust loop; however, faith is less dependent upon the partner and more whether the collaboration itself is perceived as valuable in each separate organisation. A lack of faith in an arrangement can also lead to its dissolution—but this is a dissolution that is more from intra-organisational than inter-organisational origin. Furthermore, Fig. 3 illustrates that as collaborative functioning (particularly trust) dwindles, so too does synergy and the ability for a collaboration to perform as required. This has the risk of leading to collaborative inertia, wherein the focus is entirely on maintaining and repairing the collaboration rather than achieving the aims of the collaboration itself. Only when the functioning of the collaboration is going smoothly can synergy, and thus performance, be maximised. This may explain why some collaborations do not produce better outcomes when others do—as they are stuck in the collaborative inertia of figuring out how to function rather than to perform. Of course, it is also possible for collaborations to move up and down the inertia and performance scale over time as events occur.

The CMOCs that we have identified in Table 3, where phrased either positively or negatively, in most cases, are likely to also be able to be reversed so the inverse is true: i.e. just as a shared vision can reduce conflict and enhance trust, so too can a:

*Lack of shared vision (context) ➔ increase conflict (mechanism) ➔ and reduce trust (outcome)*

This inversion is likely to be the case where contextual factors are on a continuous scale (i.e. organisational size, degree of workforce instability), or involve relatively binary cases (i.e. where a collaboration is entered into voluntarily or through mandate).

The potential impact of mandated collaboration and hierarchical structuring on trust

Other authors in the field have situated inter-organisational collaborations as a spectrum from hierarchical relations (i.e. acquisition or merger), through to market relations, in which only price mechanisms bring organisations together temporarily [94]. Our initial review of typologies of healthcare collaborations [29] identified a similar spectrum, defined by the degree of integration in terms of structure and governance, with individual buddying arrangements characterised by low integration and a full merger by high integration [94].

Some readers may consider mergers and acquisitions to not be classified as forms of inter-organisational collaborations due to their mandated nature (from the perspective of the acquired) and intention to unify as a singular entity. We agree that, after the merging is complete, the resulting organisation would count as a singular entity from a structural standpoint; however, we contend that the processes of collaboration are nonetheless key during the implementation of the merger or acquisition itself and persist for some time thereafter. We do believe that both arrangements such as buddying and mergers are compatible within this same overarching realist theory. For example, in a merger or acquisition, the organisation being acquired may perceive the ‘collaboration’ as imposed upon them (and thereby be mandated). This change in configuration of the context may change mechanisms at play from trust-building towards confidence in contractual relations [53].

**Fig. 4:**
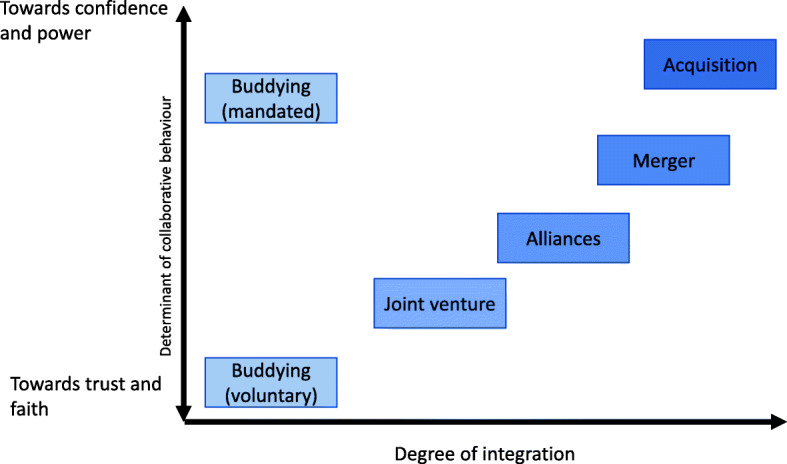
The shift from trust to confidence as a determinant for collaborative behavior in mandated and more integrative forms of collaboration

Along this spectrum of integration, then, from buddying (low) to mergers and acquisitions (high), it is likely that trust and faith as key mechanisms see a gradual shift towards power, legal frameworks, and formal control mechanisms as the primary determinant for engaging in collaborative behaviour and achieving synergy. This is due to the complexity at hand making formalisation more required, and a tendency towards more integrative forms typically being mandated (or the result of an organisational failure) (Fig. 4) [94]. This change in driver of collaborative behaviour is due to the change in reasoning around *perception of risk* (and thus trust) being the determining factor for collaborative behaviour, towards *contractual obligation* making collaborative behaviour required. Since whether a collaboration is mandated or voluntary is often rather binary comes with a rigid power structure, it is also possible for mandated forms of buddying, for example, to rely heavily on formal control mechanisms as a basis for collaborative behaviour, while still being relatively non-integrative (Fig. 4) [29]. This is reflected by sources such as McNamara (p. 70), who state that voluntary collaboration is characterised by ‘shared power arrangements’ and ‘informal and formal agreements', whereas mandated collaborations typically have ‘hierarchical arrangement with convening authority oversight’ where the ‘mandate formalises structural elements of [the] planning process’ [76]. As such, both mandated and more integrative forms of collaboration can be considered on the same side of the spectrum from a formalisation and structural perspective (Fig. 4).

Although included papers only mentioned formalisation as a context for improved trust, others in the literature have drawn on the concept of *confidence* as another means of upholding collaborative behaviour and the perception of risk [75]. Smith ( [75]; p. 308) has put forward the notion of ‘differences between morally and socially supported trust, and confidence that relies on “contract or other regulatory forms” to secure co-operation’. According to Smith (p. 309), this confidence is ‘supported by external referents’, whereas ‘trust is supported by internal referents most notably affective and moral motivation to act in a trusting and trustworthy way’ [75]. This concept of confidence may underline these findings as a realist mechanism that can support collaboration in place of trust in more integrative and mandated collaboration types, in which a clear power structure exists.

However, although organisational actors may engage in collaborative behaviour when contractually obligated to do so due to confidence, it does not mean that having a basis of trust would not also be helpful. According to our programme theories, having a base of trust and faith in the collaboration would still result in lesser conflict and more self-motivated drive to work on collaborative tasks. Our initial CMOCs for this element would read:

*Mandated or highly integrated collaboration (context) ➔ increased formalisation (mechanism) ➔ improved confidence (outcome)*

*Increased confidence (context) ➔ increased synergy (mechanism) ➔ increased collaborative performance (outcome)*

These initial CMOCs about confidence and trust will be assessed more in the testing phase of this realist synthesis.

### Implications

The present paper builds upon existing evidence by using a realist methodology, identifying initial CMOCs of how inter-organisational collaborations work in a healthcare setting. Other papers have looked at barriers or enablers of collaborations [4] and have compared factors affecting inter-organisational versus interprofessional collaboration [95], but this review builds upon these by making explicit the underlying mechanisms at play for both collaboration functioning and performance. It also incorporates other contextual elements, such as the motivations for entering into a collaboration at the outset. This provides greater insight into the complex systems nature of the many interacting elements at work between interpersonal, environmental, and macro-scale factors [4, 23]. Many of the contextual factors identified in our review in a bottom-up manner are nonetheless similar to those identified by other reviews, such as organisational size and degree of formalisation, increasing the validity of our findings [21]. However, we build upon these by drawing links between contexts and the effect they have on the reasoning and behaviour of actors involved in the intervention (i.e. the impact on trust, faith, and other mechanisms).

Our research also supports the findings of other realist reviews in the literature on related topics, e.g. those by Jagosh et al., through its similar inclusion of the trust-building loop, and we also build upon existing realist-based studies of healthcare collaboration [18, 19, 96]. While these contributions draw attention to particular intervention types and contexts, our contribution provides much needed insight into the inter-organisational entities currently being promoted across healthcare settings. These findings demonstrate that many inter-organisational collaborations may struggle to achieve the collaborative synergy required for better performance due ‘collaborative inertia’ brought about by unfavourable configuration of contexts. However, given the increasing recognition that healthcare systems will need to adapt in order to face financial challenges and ‘surge’ events such as COVID-19 [10], working together in a ‘whole-systems’ manner is needed more than ever [97]. Likewise, the need for practical advice, such as that proposed in this review, is essential for supporting inter-organisational collaboration [98].

As an example of practical application of our findings, those interested in, or currently involved with, enforced mergers, could use a combination of Fig. 3 and our CMOCs to understand that entering an enforced arrangement is likely to reduce the initial trust level, limiting the aims partners may agree to, and putting the collaboration at risk for conflict earlier on in the process than otherwise may have occurred. According to our analysis, it is clear that maximising initial trust through any means possible is essential to starting on the right foot and for increasing the chance of rapidly achieving synergy and avoiding inertia. So, changing the context through the use of stronger legal agreements to uphold roles and responsibilities, fostering a shared vision, and drawing upon prior collaborative experience, as well as putting in place robust accountability and governance arrangements in case of conflict, can all go a long way to increasing initial trust level and mitigating further loss of trust if/when conflicts occur.

Further research is required to complement the secondary and theoretical evidence from the literature. We suggest it can be further enhanced by synthesising data from primary data collection. It will be an aim of our ongoing work to test these CMOCs against real-life examples from a range of collaboration types to ensure broad applicability using data we will collect, as well as further case studies from the literature. While the CMOCs we identified are useful for understanding what works, for whom, and under what circumstances, they are less useful for establishing ‘when’ various contexts may need to be configured differently to produce the most beneficial outcomes [75]. Building upon our CMOCs with a greater understanding of ‘when’ would provide even further applicability to our review, giving implementers and evaluators an understanding of what should be implemented, how, why, and when. We will seek to expand this with the next phase of our realist synthesis.

## Limitations

It is possible that the search strategy used missed some key literature that could have added to the evidence included in this review. While the review predominately draws on UK examples of inter-organisational collaboration, the inclusion of many reviews, which themselves included many case studies, lessened the potential for missing any further contextual elements. It was clear that theoretical saturation was reached during coding of contextual factors and mechanisms. We also retained some literature for use in the next theory refinement phase of the realist synthesis, by limiting the breadth of databases and terminology we used for the search. As such, it is likely that we were not able to formulate CMOCs for every eventuality; however, further details will be explored in the next stage of realist synthesis. As our analysis is intended to cover a variety of collaboration types from buddying through mergers, as opposed to a singular form of programme, the CMOCs we proposed are constructed at a relatively high level of abstraction.

One further limitation which is broadly representative of the literature is the relative lack of information on collaborative entities such as buddying. While one case study was included, there was only minor information about contextual factors or mechanisms, as exploring these was not the main aim of their paper, and so contextual elements and mechanisms were only mentioned in passing. As such, more detailed, realist investigations of these phenomena are required. We also encountered issues determining which factors are contexts or mechanisms—which is not unheard of [79]; but, we mostly were able to identify that this largely depended upon which part of the causal and temporal chain was being analysed, examples of which is reflected in Fig. 3.

## Conclusion and recommendations

This paper used a realist review methodology, combining systematically identified literature with purposively identified theoretical papers to formulate context-mechanism-outcome configurations about how inter-organisational collaborations in healthcare work, and why. The systematic search screened 2769 titles and abstracts, resulting in 39 included papers from a varied set of literature encompassing various forms of collaboration and fourteen further purposively identified papers to inform the middle-range theories. Our findings identified that trust and conflict are essential to sustaining the functioning of a collaboration, with these mechanisms depending upon contextual elements such as cultural differences, establishment of governance and accountability arrangements, prior experiences of partnership, and geographical proximity of the involved organisations. Entry points into collaboration were also key to shaping the context, particularly whether entry was voluntary or mandatory.

Underlying collaborative performance was partnership synergy, which was only maximised when collaborative functioning (underpinned by trust, faith, and interpersonal communication) was at its peak. These understandings led to the proposal of a model which outlines how factors such as trust, risk tolerance, aim accomplishment, and conflict may interact in a cyclical manner, underpinning the ability for collaborations to achieve synergy and maximise performance. Likewise, it was found essential to avoid collaborative inertia, to maintain trust, and to manage conflict effectively to avoid dissolution. More integrative or mandated collaboration types may rely less on trust and more on confidence in formalised procedures to drive collaborative behaviour. While these configurations are based upon robust literature, the task of testing these against real-life case studies still remains. This will be conducted in the next phase of our realist synthesis. We expect these programme theories to give essential understanding to those involved in implementing, evaluating, or proposing collaborations between healthcare organisations.

## Supplementary Information

**Additional file 1.** Contextual factors that affect how mechanisms are activated.

**Additional file 2.** Search strategies.

**Additional file 3.** RAMESES Reporting Standard Checklist.

## Data Availability

The datasets generated and analysed during this study are available from the corresponding author on reasonable request.

## Abbreviations

CMOC: Context-mechanism-outcome configuration
HMIC: Healthcare Management Information Consortium
NHS: National Health Service
NIHR: National Institute for Health Research
MRT: Middle-range theory
PRISMA: Preferred Reporting Items for Systematic Reviews and Meta-Analyses
PROSPERO: International Prospective Register of Systematic Reviews
RAMESES: Realist And Meta-narrative Evidence Syntheses: Evolving Standards
UK: United Kingdom
