# Supplementary material for: Why do some inter-organisational collaborations in healthcare work when others do not? A realist review
Source: Syst Rev. 2021 Mar 22;10:82. doi: 10.1186/s13643-021-01630-8 (PMC7984506; doi:10.1186/s13643-021-01630-8)
Supplement: Supplementary file 1 — Additional file 1. Contextual factors that affect how mechanisms are activated. [file 13643_2021_1630_MOESM1_ESM.docx]

| Contextual factor | Explanation | Sources | Posited mechanism |
| --- | --- | --- | --- |
| Respect | An aspect of trust – how much a partner values the other | Dowling et al (2004); Lewis (2005); Round & Ashworth (2018); Cameron et al (2014) | Trust |
| Interpersonal communication | Frequency and quality of dialogue between partners | Auschra (2018); Hunter & Perkins (2012); Casey (2008) | Trust |
| Former collaboration experience | Whether partners have former collaborative experiences, whether these were positive or negative | Auschra (2018); Round & Ashworth (2018) | Trust |
| Formalisation | To what degree organisational actions are enshrined in contractual form | Casey (2008); Mandell & Steelman (2003) | Trust, confidence |
| Trust and Risk | Trust allows for taking on a risk by engaging in a collaborative endeavour. Depends upon expectations or stereotypes about the other party | Auschra (2018); Zuckerman (1995); Murray et al (2018); Gannon-Leary et al. (2006); Adedoyin et al (2019); Ball et al (2010); Dowling et al (2004); Hudson et al (1999); Hunter & Perkins (2012); Huxham (2003); Round & Ashworth (2018); Wildridge et al (2004); Zuckerman (1995); Casey (2008); Cameron et al (2014); NHS Professionals (2019) | Trust |
| Shared vision | How the partners see the nature of their relationship | Adedoyin et al (2019); Dowling et al (2004); Evans & Killoran (2000); Gannon-Leary et al. (2006); Hudson et al (1999); The King’s Fund (2005); Wildridge et al (2004); Casey (2008); Cameron et al (2014) | Trust |
| Historical context in area | Whether other organisations in the area have a history of collaboration and how this causes stereotypes in the minds of other organisations nearby | Auschra (2018) | Trust |
| Focus on outcomes | Focusing on long vs. short term outcomes | Zuckerman (1995); Hunter & Perkins (2012) | Trust |
| Ambition | Whether the aims and objectives of the partnership are realistic given the resources available for the task | Dickinson & Glasby (2010); Round & Ashworth (2018); Gannon-Leary et al. (2006); Hudson et al (1999); Lim (2014); Shaw (2002) | Faith, trust |
| Existing cooperative networks | The experiences involved partners have had with prior collaborations | Evans & Killoran (2000); Hudson et al (1999); Wildridge et al (2004); Mandell & Steelman (2003); Cameron et al (2014) | Trust |
| Type of aims/objectives/problem to solve/motivation for partnership | The type of problem that collaboration is intended to rectify, and how complex it is | Mandell & Steelman (2003) | Initial trust level |
| Power | The relative size, reputation, or other aspect of an organisation is greater in one than another, causing decision-making to be unequal | Auschra (2018); Round & Ashworth (2018); Zuckerman (1995); Cereste et al (2003); Fulop et al (2002); Mandell & Steelman (2003); Casey (2008) | Conflict and trust |
| Willingness to change (workforce and leadership) | How entrenched the actors are in their existing, pre-collaboration mindsets | Auschra (2018); Casey (2008) | Conflict |
| Culture | Norms, attitudes, and behaviours of workforces of organisations | Auschra (2018); Round & Ashworth (2018); Murray et al (2018); Gannon-Leary et al. (2006); Cameron et al (2014); Shaw (2002); NHS Professionals (2019) | Conflict |
| Organisational vs collective interests | Balancing the aims of one’s organisation vs. those of the collaboration, especially as they may conflict | Auschra (2018); Hunter & Perkins (2012); Casey (2008); Peck, Towell & Gulliver (2001) | Conflict |
| Dissolving partnership as appropriate | Whether the partnership is dissolved once its aims have been met | Hunter & Perkins (2012) | Conflict |
| Task delegation/clarity of roles | How effectively tasks are delegated to the right actors/partner – linked to how clear and defined roles are | Douglas (1998); The King’s Fund (2005); Cameron et al (2014); Hudson et al (1999); Hunter & Perkins (2012) | Conflict, Perception of progress |
| Appropriate structures, accountability | Whether the appropriate governance structures are put into place to oversee the partnership | Dowling et al (2004); The King’s Fund (2005); Wildridge et al (2004); Evans & Killoran (2000); Hudson et al (1999); Hunter & Perkins (2012); Leach et al (2019); NHS Professionals (2019); Billings et al (2015); Casey (2008) | Conflicts |
| Pace of development | How rapidly the partnership is implemented and is up and running | Wildridge et al (2004); Billings et al (2015); What Works Scotland (2015); Evans & Killoran (2000); Ferrier & Valdmanis (2004) | Conflict |
| Organisational ownership | How committed the actors are to the partnership and to what extent they feel responsibility for the process | Evans et al (2016); Hudson et al (1999); Hunter & Perkins (2012); Leach et al (2019); Wildridge et al (2004); Mandell & Steelman (2003) | Conflict |
| Management of shared resources | How fairly shared resources are managed, linked to power | Douglas (1998); Cameron et al (2014) | Power |
| Congruence of aims & objectives | To what extent partners agree on their aims and objectives – may be affected by power | Auschra (2018); Evans & Killoran (2000); Dickinson & Glasby (2010); Round & Ashworth (2018); Zuckerman (1995); Murray et al (2018); Adedoyin et al (2019); Ball et al (2010); Gannon-Leary et al. (2006); Huxham (2003); The King’s Fund (2005); Dowling et al (2004); Wildridge et al (2004); Casey (2008); Cameron et al (2014) | Conflict, power |
| Involvement of lower-level staff | Whether lower-level managers and members of the workforce are involved in decision-making around the partnership | Zuckerman (1995); Cameron et al (2014); Idel (2003) | Intra-organisational conflict, faith |
| Workforce stability | Whether employees are lost or retained during the partnership process | Hunter & Perkins (2012); Casey (2008); Idel (2003); Lim (2014); Shaw (2002) | Intra-organisational conflict, trust, faith |
| Ongoing evaluation | How well the partnership is evaluated and required changes made during and after implementation | Ball et al (2010); Douglas (1998); Hunter & Perkins (2012); Round & Ashworth (2018); Billings et al (2015 | Perception of progress |
| Engagement of stakeholders | To what extent stakeholders are involved in the partnership process, creation of aims, evaluation, etc. | Ball et al (2010); Cereste et al (2003); Evans & Killoran (2000); Evans et al (2016); Kendall et al (2012); Round & Ashworth (2018); Billings et al (2015); Mandell & Steelman (2003); Starling (2018); Wildridge et al (2004) | Perception of progress |
| Direction of focus | Focusing on high vs. low priority tasks | Round & Ashworth (2018) | Perception of progress |
| Readiness for change/organisational flexibility | How well and rapidly an organisation is able to adapt to change – as is required with a partnership | Evans & Killoran (2000); Evans et al (2016); Hudson et al (1999); Kendall et al (2012); What Works Scotland (2015); Wildridge et al (2004); Cameron et al (2014) | Perception of progress |
| Presence of actors | Whether the staff whose knowledge and skills are required to implement the partnership are accessible | Auschra (2018) | Perception of progress |
| Financial resource | How much financial capacity there is for implementing partnership activities and structures | Auschra (2018); Round & Ashworth (2018); What Works Scotland (2015) | Perception of progress |
| Leadership/management | Whether leaders have the appropriate knowledge, and use the appropriate skills, to manage the partnership | Ball et al (2010); Douglas (1998); Evans & Killoran (2000); Hearld et al (2015); Hunter & Perkins (2012); Leach et al (2019); Starling (2018); Murray et al (2018); Wildridge et al (2004); Cameron et al (2014) | Conflict, faith |
| Authenticity of partnership | Whether the partnership is genuine and stakeholder-focused, or done to look trendy | Dickinson & Glasby (2010); Hunter & Perkins (2012) | Faith |
| Geography | Proximity of organisations to each other | Auschra (2018); Round & Ashworth (2018); Kershaw et al (2018); Cameron et al (2014) | Interpersonal communication/coordination |
| Information exchange and IT systems | How well information can be exchanged, usually depends upon IT systems | Auschra (2018); Hunter & Perkins (2012); Adedoyin et al (2019); Evans et al (2016); Cameron et al (2014); NHS Professionals (2019) | Interpersonal communication/coordination |
| Regulatory environment | The impact of governmental (and other organisation’s) policy on the ease of collaborating | Auschra (2018); Round & Ashworth (2018); Mandell & Steelman (2003) | Task complexity |
| Organisational size | Relative sizes of the organisations involved in the partnership | Gaynor et al (2012); Fulop et al (2002) | Task complexity |
| Partnership synergy | A context of synergy allows for maximal partnership performance & accomplishment of desired outcomes | Dickinson, Peck, and Davidson (2007; Huxham (2003); Auschra (2018); Kendall et al. (2012); Glasby and Dickinson (2009); Casey (2008); Gannon-Leary, Baines, and Wilson (2006); Ball et al. (2010) | Partnership performance, Perception of progress |
| Collaborative inertia | Getting stuck in the daily running of the collaboration distracts manpower from achieving the aims of the actual partnership | Huxham (2003); NHS Professionals (2019); Dickinson, Peck, and Davidson (2007); Hudson et al. (1999); Casey (2008) | Partnership performance, Perception of progress |
