# Supplementary material for: Why do some inter-organisational collaborations in healthcare work when others do not? A realist review
Source: Syst Rev. 2021 Mar 22;10:82. doi: 10.1186/s13643-021-01630-8 (PMC7984506; doi:10.1186/s13643-021-01630-8)
Supplement: Supplementary file 2 — Additional file 2. Search strategies. [file 13643_2021_1630_MOESM2_ESM.docx]

# Search strategies

HMIC Commentary search strategy

1. buddying.mp.

2. (clinical adj1 network).mp. [mp=title, other title, abstract, heading words]

3. merger.mp.

4. acquisition.mp.

5. (joint adj1 management).mp. [mp=title, other title, abstract, heading words]

6. consolidation.mp.

7. (coordinating or co-ordinating or coordination or co-ordination).mp. [mp=title, other title, abstract, heading words]

8. (hospital adj1 chain$).mp. [mp=title, other title, abstract, heading words]

9. federation.mp.

10. (joint adj1 working).mp. [mp=title, other title, abstract, heading words]

11. (partnership adj1 working).mp. [mp=title, other title, abstract, heading words]

12. alliance.mp.

13. (joint adj1 commissioning).mp. [mp=title, other title, abstract, heading words]

14. vanguard.mp.

15. exp Integration/

16. 1 or 2 or 3 or 4 or 5 or 6 or 7 or 8 or 9 or 10 or 11 or 12 or 13 or 14 or 15

17. health care/ or acute care/ or medical care/ or primary care/ or public sector/

18. 16 and 17

19. opinion*.ti.

20. (view or views).ti.

21. editorial.mp.

22. note.mp.

23. comment.mp.

24. letter.mp.

25. or/19-24

26. 18 and 25

27. limit 26 to yr="1990"

HMIC review, framework, theory, evaluation search strategy

1 buddying.mp.

2 (clinical adj1 network).mp. [mp=title, other title, abstract, heading words]

3 merger.mp.

4 acquisition.mp.

5 (joint adj1 management).mp. [mp=title, other title, abstract, heading words]

6 consolidation.mp.

7 (coordinating or co-ordinating or coordination or co-ordination).mp. [mp=title, other title, abstract, heading words]

8 (hospital adj1 chain$).mp. [mp=title, other title, abstract, heading words]

9 federation.mp.

10 (joint adj1 working).mp. [mp=title, other title, abstract, heading words]

11 (partnership adj1 working).mp. [mp=title, other title, abstract, heading words]

12 alliance.mp.

13 (joint adj1 commissioning).mp. [mp=title, other title, abstract, heading words]

14 vanguard.mp.

15 exp Integration/

16 health care/ or acute care/ or medical care/ or primary care/

17 administration/ or organisational structure/ or organisational theory/ or organisations/

18 15 or 16 or 17

19 7 or 10

20 18 and 19

21 1 or 2 or 3 or 4 or 5 or 6 or 8 or 9 or 11 or 12 or 13 or 14 or 20

22 evaluat$.mp.

23 interven$.mp. [mp=title, other title, abstract, heading words]

24 assess$.mp. [mp=title, other title, abstract, heading words]

25 trial.mp. [mp=title, other title, abstract, heading words]

26 model.mp. [mp=title, other title, abstract, heading words]

27 review.mp. [mp=title, other title, abstract, heading words]

28 (case adj1 study).mp. [mp=title, other title, abstract, heading words]

29 theory.mp. [mp=title, other title, abstract, heading words]

30 22 or 23 or 24 or 25 or 26 or 27 or 28 or 29

31 21 and 30

32 limit 31 to yr="1990 -Current"

OVID – Medline and PsycINFO search strategy

1 buddying.mp.

2 (clinical adj1 network).mp.

3 merger.ti.

4 acquisition.ti.

5 (joint adj1 management).mp.

6 consolidation.ti.

7 (coordinating or co-ordinating or coordination or co-ordination).ti.

8 (hospital adj1 chain$).ti.

9 federation.ti.

10 (joint adj1 working).ti.

11 (partnership adj1 working).mp.

12 alliance.ti.

13 (joint adj1 commissioning).ti.

14 vanguard.mp.

15 systems integration/ or "health care facilities, manpower, and services"/ or "health care economics and organizations"/ or health services administration/ or "health care quality, access, and evaluation"/

16 partnership.mp.

17 partnering.mp.

18 1 or 2 or 3 or 4 or 5 or 6 or 7 or 8 or 9 or 10 or 11 or 12 or 13 or 14 or 16 or 17

19 15 and 18

20 limit 19 to yr="1990 -Current"

Social policy and practice database search strategy

1 partnering.mp. [mp=abstract, title, publication type, heading word, accession number]

2 partnership.mp. [mp=abstract, title, publication type, heading word, accession number]

3 "joint working".mp. [mp=abstract, title, publication type, heading word, accession number]

4 merger.mp. [mp=abstract, title, publication type, heading word, accession number]

5 acquisition.mp. [mp=abstract, title, publication type, heading word, accession number]

6 alliance?.mp. [mp=abstract, title, publication type, heading word, accession number]

7 "partnership working".mp. [mp=abstract, title, publication type, heading word, accession number]

8 buddying.mp. [mp=abstract, title, publication type, heading word, accession number]

9 (clinical adj1 network).mp. [mp=abstract, title, publication type, heading word, accession number]

10 (coordinating or co-ordinating or coordination or co-ordination).mp. [mp=abstract, title, publication type, heading word, accession number]

11 (joint adj1 commissioning).mp. [mp=abstract, title, publication type, heading word, accession number]

12 vanguard.mp. [mp=abstract, title, publication type, heading word, accession number]

13 integration.mp. [mp=abstract, title, publication type, heading word, accession number]

14 healthcare.mp. [mp=abstract, title, publication type, heading word, accession number]

15 hospital?.mp. [mp=abstract, title, publication type, heading word, accession number]

16 evaluat*.mp. [mp=abstract, title, publication type, heading word, accession number]

17 intervention.mp. [mp=abstract, title, publication type, heading word, accession number]

18 model.mp. [mp=abstract, title, publication type, heading word, accession number]

19 review.mp. [mp=abstract, title, publication type, heading word, accession number]

20 (case adj1 study).mp. [mp=abstract, title, publication type, heading word, accession number]

21 theory.mp. [mp=abstract, title, publication type, heading word, accession number]

22 1 or 2 or 3 or 4 or 5 or 6 or 7 or 8 or 9 or 10 or 11 or 12 or 13

23 14 or 15

24 16 or 17 or 18 or 19 or 20 or 21

25 22 and 23 and 24
